# Conservation and divergence in NaChBac and Na_V_1.7 pharmacology reveals novel drug interaction mechanisms

**DOI:** 10.1038/s41598-020-67761-5

**Published:** 2020-07-01

**Authors:** Wandi Zhu, Tianbo Li, Jonathan R. Silva, Jun Chen

**Affiliations:** 1 Biochemical and Cellular Pharmacology, Genentech Inc., 103 DNA Way, South San Francisco, CA USA; 2 Biomedical Engineering, Washington University in St. Louis, St. Louis, MO USA

**Keywords:** Biophysics, Ion transport, Electrophysiology, Patch clamp, Pharmacology, High-throughput screening

## Abstract

Voltage-gated Na^+^ (Na_V_) channels regulate homeostasis in bacteria and control membrane electrical excitability in mammals. Compared to their mammalian counterparts, bacterial Na_V_ channels possess a simpler, fourfold symmetric structure and have facilitated studies of the structural basis of channel gating. However, the pharmacology of bacterial Na_V_ remains largely unexplored. Here we systematically screened 39 Na_V_ modulators on a bacterial channel (NaChBac) and characterized a selection of compounds on NaChBac and a mammalian channel (human Na_V_1.7). We found that while many compounds interact with both channels, they exhibit distinct functional effects. For example, the local anesthetics ambroxol and lidocaine block both Na_V_1.7 and NaChBac but affect activation and inactivation of the two channels to different extents. The voltage-sensing domain targeting toxin BDS-I increases Na_V_1.7 but decreases NaChBac peak currents. The pore binding toxins aconitine and veratridine block peak currents of Na_V_1.7 and shift activation (aconitine) and inactivation (veratridine) respectively. In NaChBac, they block the peak current by binding to the pore residue F224. Nonetheless, aconitine has no effect on activation or inactivation, while veratridine only modulates activation of NaChBac. The conservation and divergence in the pharmacology of bacterial and mammalian Na_V_ channels provide insights into the molecular basis of channel gating and will facilitate organism-specific drug discovery.

## Introduction

Electrical signaling is a highly conserved biological mechanism throughout the evolution of plants and animals^1,2^. From prokaryotic organelles to vertebrates, ion channels are key regulators of cellular homeostasis, electrolyte balance and signaling^1^. Single-celled eukaryotes first evolved voltage-gated calcium channels (Ca_V_), which are believed to have evolved subsequently into voltage-gated Na^+^ (Na_v_) channels predating the origin of nervous systems in animals^3^. In prokaryotes, Na_V_ channels might have evolved independently to regulate cellular homeostasis, though their exact functions are not well understood.

In humans, the malfunction of Na_V_ channels, either through genetic mutations or off-target drug interactions, results in severe pathologies including cardiac arrhythmia and epilepsy^4^. Given their critical physiological function, pathological relevance and utility as therapeutic targets, it is essential to understand the interaction of Na_V_ channels with pharmacological agents. This task has been facilitated by the recent determination of prokaryotic^5–7^ and eukaryotic^8–13^ Na_V_ channel structures. However, these structures do not provide a clear picture of the dynamic conformational changes that underlie state and voltage-dependent effects of pharmacological agents. Therefore, functional studies must be integrated with structural insights to elucidate the molecular mechanisms of compound-channel interaction^14,15^.

Eukaryotic Na_V_ channels are large, complex membrane-bound proteins composed of four homologous domains that are connected via long intracellular linkers^16^. In contrast, prokaryotic Na_V_ channels are formed by co-assembly of four identical subunits^6,7^. Bacterial Na_V_ channels are useful as model systems for their more complex eukaryotic counterparts because their crystal structures in various states have been solved, providing unprecedented insights into ion selectivity, gating, as well as drug interaction mechanisms^5–7,17–19^. For example, the crystal structure of Na_V_Ab (from *Arcobacter butzleri*) with lidocaine and flecainide reveals that the potency of resting state block by these local anesthetics (LAs) is determined by the size of fenestrations that connect the lipids in the cell membrane with the inner pore of the channel, the so-called hydrophobic pathway for drug entry^20^. This notion is corroborated by pharmacological characterization, as lidocaine and benzocaine block NaChBac channel when applied extracellularly, suggesting the conservation of this hydrophobic pathway in related channels. Despite such progress, the pharmacology of bacterial voltage-gated Na^+^ (BacNa_V_) channels remains largely underexplored.

In this study, we systematically investigate the interaction of NaChBac (from *Bacillus* halodurans) with 39 Na^+^ channel modulators from various drug classes. Comparing the pharmacology of NaChBac with human Na_V_1.7 channels reveals that while some drug interaction mechanisms are conserved, others exhibit striking divergence.

## Results

### Effects of local anesthetic (LA) site-binding small molecules on the NaChBac channel

**Figure 1:**
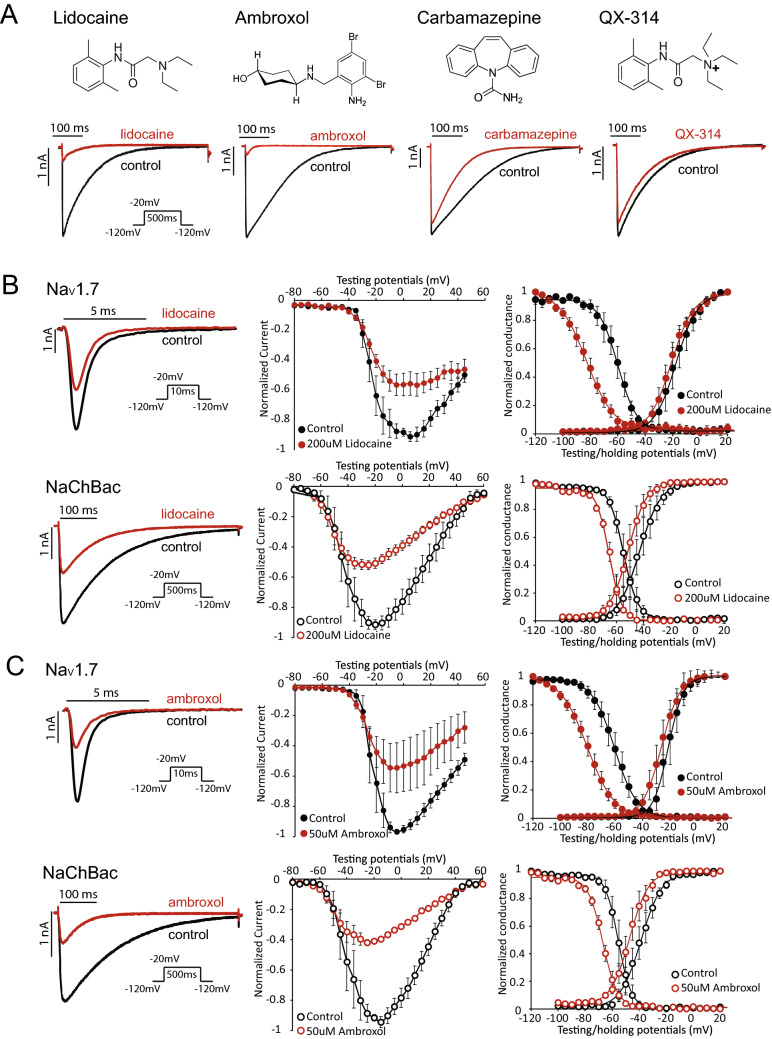
The local anesthetics lidocaine and ambroxol differently modulate Na_V_1.7 and NaChBac channel gating. (**A**) Chemical structures of four selected small molecule pore blockers, ambroxol, lidocaine, carbamazepine, and QX-314. Representative NaChBac current traces are shown before and after the application of compounds. Testing concentrations for the compounds are shown in Supplement Table I. (**B**) Lidocaine’s effect on the Na_V_1.7 (top) and NaChBac (bottom) channels. Current traces (left), current–voltage (I–V) (middle), conductance–voltage (G–V), and steady-state inactivation (SSI) relationships are shown for both Na_V_1.7 and NaChBac measured before and after application of 200 µM lidocaine. Lidocaine blocks both NaChBac and Na_V_1.7 channels. (**C**) Ambroxol’s effect on Na_V_1.7 (top) and NaChBac (bottom) channels. Representative current traces (left), I-V (middle), G–V, and SSI relationships (right) are shown for Na_V_1.7 and NaChBac channels measured before and after 50 µM ambroxol. Ambroxol has similar effects as lidocaine on both Na_V_1.7 and NaChBac.

A group of 16 small molecule compounds known to block mammalian Nav channels, including antiarrhythmics, anticonvulsants, muscle relaxants, and local anesthetics were selected for evaluation. To carry out the initial screening, compounds were tested at a concentration that was 50% higher than the known IC_50_ values for the least-sensitive mammalian Na_V_ channel isoform. When tested against NaChBac channels, 11 out of 16 compounds caused robust block, whereas five compounds, carbamazepine, oxcarbazepine, co102862, QX-222 and QX-314 were relatively ineffective, resulting in only 15–31% inhibition (Fig. 1A, Supplement Fig. 1A, Supplement Table I). Among the inactive compounds, QX-222 and QX-314 are permanently charged. Our results are consistent with a previous report that QX-314 has no effect on the NaChBac channel when applied extracellularly^21^. In contrast to QX-222 and QX-314, the anticonvulsants carbamazepine, oxcarbazepine, and co102862 are highly hydrophobic and are almost completely neutral at physiological pH. Thus, a majority of LA site-binding small molecules block NaChBac channels, with the exception of the positively charged or highly hydrophobic compounds.

We further examined two of the ‘hit’ compounds (defined by > 40% block at test concentration), lidocaine and ambroxol on NaChBac. In response to 200 µM lidocaine, both Na_V_1.7 and NaChBac exhibited a similar level of tonic block, as shown in representative current traces and current–voltage (I–V) relationships (Fig. 1B). Lidocaine had a minimal effect on the voltage-dependence of activation (G–V relationship) of Na_V_1.7, but significantly shifted the steady-state inactivation (SSI) curve to more negative potentials (Fig. 1B, top right, ΔV_1/2_ = − 22.9 ± 2.0, p = 0.0003). These effects were described previously^22–24^ and indicate that lidocaine stabilizes Na_V_1.7 channels in an inactivated conformation. Lidocaine induced a smaller hyperpolarizing shift in the SSI curve of NaChBac channel currents (Fig. 1B, bottom right, ΔV_1/2_ = − 11.1 ± 3.2.0, p = 0.007). Lidocaine also increased the slope factor (*k[n]*) of the SSI curve of Na_V_1.7, an effect not observed for NaChBac (Table 1). This difference may be explained by differences in the mechanism of inactivation for the two channel types. NaChBac channel lacks the fast inactivation gate in Na_V_1.7 channel, hence its inactivation might be mediated by an alternative and slower mechanism, such as pore collapse.

**Table 1:** Parameters of Boltzmann fit to G–V and SSI curves for NaChBac and Na_V_1.7 channel before and after compound treatment.

	NaChBac				Na_V_1.7			
	Before lidocaine	After lidocaine	Before ambroxol	After ambroxol	Before lidocaine	After lidocaine	Before ambroxol	After ambroxol
**G–V**								
V_1/2_	− 41.8 ± 5.0	− 50.7 ± 3.7	− 38.5 ± 4.5	− 46.1 ± 4.2	− 17.6 ± 4.6	− 21.0 ± 2.7	− 21.2 ± 3.9	− 26.2 ± 3.3
k [n]	4.5 ± 0.6	4.8 ± 0.4	5.1 ± 0.7	5.7 ± 0.6	7.3 ± 0.7	7.8 ± 0.2	5.0 ± 0.7	6.7 ± 0.2
**SSI**								
V_1/2_	− 54.3 ± 2.7	− 65.4 ± 1.7	− 55.1 ± 2.8	− 66.7 ± 1.5	− 60.0 ± 2.6	− 82.9 ± 2.4	− 60.4 ± 3.7	− 80.3 ± 2.7
k [n]	− 4.0 ± 0.2	− 4.1 ± 0.4	− 4.0 ± 0.3	− 5.1 ± 0.4	− 6.8 ± 0.8	− 9.9 ± 0.6	− 7.9 ± 0.5	− 9.7 ± 0.2

	NaChBac				Na_V_1.7			
	Before GSAF-I	After GSAF-I	Before BDS-I	After BDS-I	Before GSAF-I	After GSAF-I	Before BDS-I	After BDS-I
**G–V**								
V_1/2_	− 49.8 ± 5.2	− 50.4 ± 7.3	− 55.3 ± 4.2	− 38.8 ± 3.3	− 18.8 ± 5.0	− 13.6 ± 3.7	− 20.2 ± 1.7	− 21.4 ± 0.1
k [n]	3.5 ± 1.2	3.7 ± 1.4	4.4 ± 0.2	5.1 ± 0.9	7.4 ± 0.9	10.5 ± 0.3	7.1 ± 0.4	3.6 ± 1.0
**SSI**								
V_1/2_	− 58.3 ± 5.5	− 63.7 ± 3.1	− 65.1 ± 4.4	− 50.8 ± 1.2	− 58.1 ± 2.7	− 64.8 ± 1.8	− 60.0 ± 1.1	− 45.8 ± 2.0
k [n]	− 3.4 ± 0.2	− 3.1 ± 0.9	− 3.5 ± 0.1	− 3.0 ± 0.2	− 6.3 ± 0.3	− 10.0 ± 0.3	− 6.6 ± 0.4	− 7.3 ± 0.2

	NaChBac				Na_V_1.7			
	Before aconitine	After aconitine	Before veratridine	After veratridine	Before aconitine	After aconitine	Before veratridine	After veratridine
**G–V**								
V_1/2_	− 54.2 ± 1.2	− 52.0 ± 1.5	− 52.4 ± 2.1	− 44.7 ± 2.0	− 19.8 ± 4.3	− 34.9 ± 1.0	− 21.0 ± 4.5	− 19.0 ± 3.0
k [n]	4.3 ± 0.3	5.0 ± 0.2	4.3 ± 0.3	10.3 ± 0.3	5.0 ± 0.8	6.7 ± 0.2	5.2 ± 0.5	5.9 ± 0.4
**SSI**								
V_1/2_	− 63.8 ± 3.7	− 66.4 ± 2.1	− 64.2 ± 3.0	− 71.4 ± 1.5	− 59.0 ± 2.4	− 65.1 ± 1.8	− 60.3 ± 2.2	− 80.5 ± 2.5
k [n]	− 3.7 ± 0.2	− 4.2 ± 0.1	− 3.7 ± 0.5	− 5.6 ± 0.2	− 6.5 ± 0.2	− 7.7 ± 0.3	− 6.4 ± 0.3	− 12.9 ± 0.4

The V_1/2_ and slope factor k[n] are shown. The control and testing extracellular recording solutions for toxins contain 0.1% BSA. We noticed a hyperpolarization shift in V_1/2_ for GV and SSI as a result of BSA.

Similar to lidocaine, 200 µM ambroxol showed robust inhibition of both Na_V_1.7 and NaChBac channels (Fig. 1C). Ambroxol induced a significant hyperpolarizing shift in the SSI curve of Na_V_1.7 (Fig. 1C, top right, ΔV_1/2_ = − 19.9 ± 2.9, p = 0.001), but the effect was not as pronounced in NaChBac (Fig. 1C, bottom right, ΔV_1/2_ = − 11.6 ± 3.5, p = 0.03). Overall, although lidocaine and ambroxol exhibit similar blockade of NaChBac and Na_V_1.7 channels, they modulate the inactivation process of these channels to different extents.

### Effect of voltage sensing domain (VSD)-binding toxins on NaChBac channels

The LA binding site resides in the pore domain, with highly conserved primary amino acid sequences across mammalian and bacterial Na_V_ channels^21^. Unlike the pore domain, the sequence of the voltage sensing domains (VSDs) exhibits much higher variability. As a result, peptide toxins that bind to the VSDs exhibit higher channel specificity when compared to LA compounds^25^. Since the effect of VSD-binding toxins on NaChBac has not been examined systematically, we tested 15 such toxins on the amplitude and gating of NaChBac channel currents at concentrations known to alter gating of mammalian Na_V_ channels. 11 toxins did not show significant effect, whereas four peptide toxins exhibited modulation on the NaChBac channel, including GsAF-I, GrTx1, GsAF-II, and BDS-I (Supplement Fig. 1B). GsAF-I, GrTx1, and GsAF-II are categorized into site 4 toxins and bind to the VSD of domain II (DII-VSD), whereas BDS-I is a site 3 toxin and interacts with the VSD of domain IV (DIV-VSD) of mammalian Na_V_ channels^26^.

**Figure 2:**
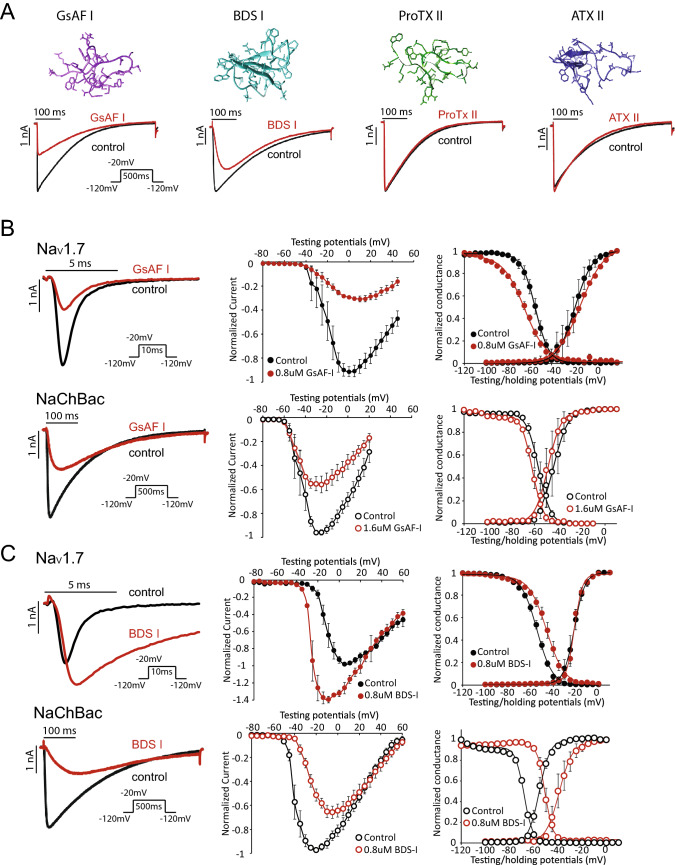
Site 3 and Site 4 VSD-binding toxins can exert unique effects on the NaChBac channel. (**A**) Simulated structure of two site 4 toxins, GsAF-I and ProTXII, and two site 3 toxins, BDS-I and ATXII (top). Representative current traces of NaChBac are shown before and after the toxin application (bottom). GsAF-I and BDS-I blocked Na^+^ conductance, while ProTXII and ATXII did not. Concentrations of the compounds are shown in Supplement Table I. (**B**) Effect of GsAF-I on Na_V_1.7 and NaChBac. GsAF-I (0.8 µM) inhibited Na_V_1.7 peak current by ~ 50%. GsAF-I (1.6 µM) inhibited NaChBac peak current by ~ 50%. Representative current traces (left), I–V (middle), G–V and SSI (right) relationships are shown before (black traces and symbols) and after GsAF-I application (red traces and symbols). (**C**) Representative current traces (left), I–V (middle), G–V and SSI (right) relationships are shown for Na_V_1.7 and NaChBac channels before (black traces and symbols), and after BDS-I (red traces and symbols). BDS-I blocked NaChBac, but augmented Na_V_1.7 current.

We chose the site 4 toxin GsAF-I and site 3 toxin BDS-I for further analysis, because these two toxins were potent inhibitors of NaChBac channels. In contrast, ProTx-II (site 4) and ATX-II (site 3) had no effect on current amplitude or kinetics of NaChBac currents (Fig. 2A). GsAF-I blocks the peak NaChBac currents and induced small shifts in the voltage dependence of inactivation (SSI) (Fig. 2B, bottom and Table 1). The overall effect of GsAF-I on Na_V_1.7 is similar to NaChBac, except in Na_V_1.7, GsAF-I caused a greater inhibition of peak currents and a shallower SSI curve (*k[n]*_control_ = − 6.3 ± 0.3, *k[n]*_GsAF-I_ = − 10 ± 0.3) (Fig. 2B, top), suggesting that in the presence of GsAF-I, more channels start to inactivation at very negative potentials.

BDS-I is thought to bind to DIV-VSD of Na_V_1.7, the VSD that is essential for controlling fast inactivation^27–29^. BDS-I increased peak Na_V_1.7 channel current at test potentials <  + 20 mV, slowed its rate of inactivation and induced a depolarizing shift in the SSI curve, but did not alter activation gating (Fig. 2C, top). In contrast, BDS-I decreased peak NaChBac channel currents at test potentials <  + 20 mV and slowed the rate of current activation (Fig. 2C, bottom). In addition, BDS-I induced a similar positive shift in both the voltage-dependence of activation (Fig. 2C, bottom right, ΔV_1/2_ = 16.5 ± 3.8 mV, p = 0.001), and SSI (ΔV_1/2_ = 14.3 ± 2.4 mV, p = 0.006), perhaps reflecting the tight coupling between channel activation and inactivation. Thus, while GsAF-I and BDS-I both inhibit peak NaChBac currents, only BDS-I markedly alters the gating of these channels.

### Lack of effects of isoform-specific compounds on NaChBac channels

Similar to the VSD-targeting toxins, isoform-specific compounds including G0766 (PF-771), G4936 (GX-936)^30^ and A803467^31^ have high specificities, as they do not bind to the common LA binding sites^30,31^. It’s not surprising that they showed no effects on the NaChBac channel (Supplement Fig. 1D and Supplement Table I), suggesting these compounds’ binding sites are not conserved in NaChBac.

### Distinct modulations of Na_V_1.7 and NaChBac by pore-binding toxins

**Figure 3:**
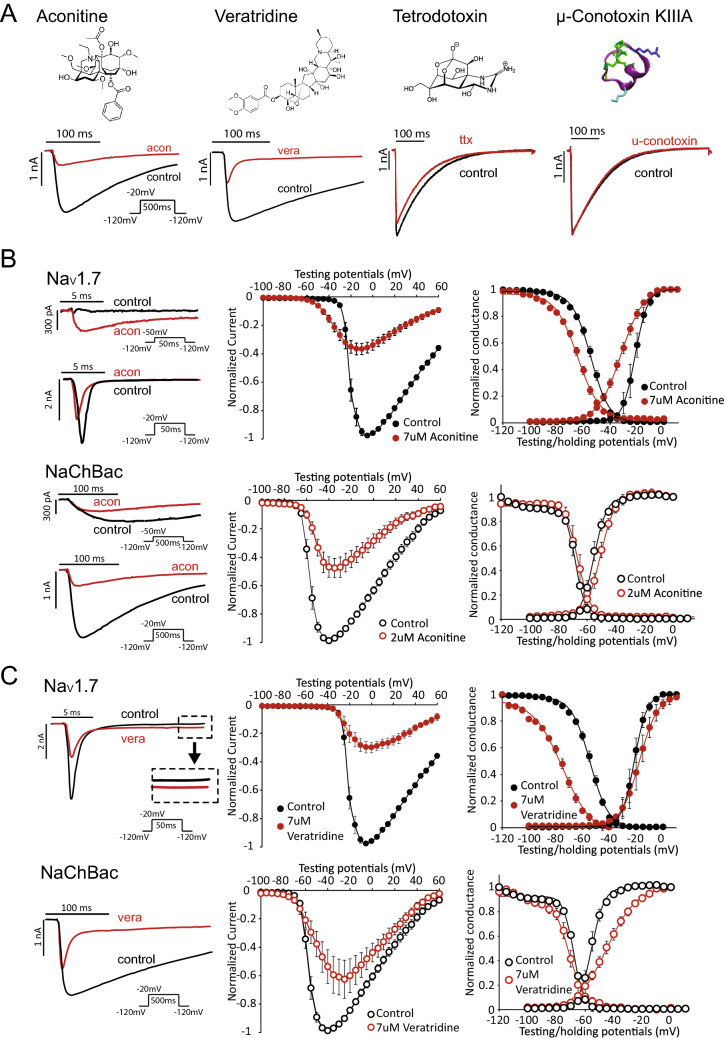
Pore binding toxins aconitine and veratridine differentially modulate Na_V_1.7 and NaChBac. (**A**) Structures of four toxins that are known to bind to the pore domain of eukaryotic Na_V_ channels. Representative current traces are shown before and after toxin application. Aconitine and veratridine, which are site-2 toxins, showed robust block of the NaChBac channel. In contrast, site 1 toxins, tetrodotoxin and µ-conotoxin KIIIA exert minimal block on NaChBac. Concentrations of the compounds are shown in Supplement Table I. (**B**) Aconitine (7 µM and 2 µM) were used to achieve ~ 50% block of the Na_V_1.7 and NaChBac channels, respectively. To illustrate the voltage-dependent response to aconitine, two sets of representative current traces are shown for both Na_V_1.7 and NaChBac. The top set is elicited by depolarizing pulse from − 120 to − 50 mV. The bottom set shows the response to a larger depolarizing pulse from − 120 to − 20 mV. The I–V, G–V, and SSI relationships are presented for Na_V_1.7 (top) and NaChBac (bottom) before and after aconitine application. (**C**) Effect of 7 µM veratridine on NaChBac and Na_V_1.7. Representative current traces before and after veratridine are shown. Inset for Na_V_1.7 current highlights the effect of veratridine on sustained Na^+^ current. I–V, G–V, and SSI relationships are also presented to quantify peak inward currents and voltage dependence of steady-state gating.

We tested several toxins that are known to bind to the pore domain of mammalian Na^+^ channels, including tetrodotoxin (TTX), u-conotoxins KIIIA, GIIIB, aconitine and veratridine, on the NaChBac channel. The site 1 toxins TTX and u-conotoxins KIIIA and GIIIB did not significantly alter currents of NaChBac, whereas the site 2 toxins aconitine and veratridine significantly blocked peak inward Na^+^ current of NaChBac (Fig. 3A and Supplement Fig. 1C). Aconitine and veratridine exhibit dual effects on the mammalian Na^+^ channels, modulating their gating to increase currents in addition to inhibiting peak Na^+^ conductance^32,33^. We assessed the effects of aconitine and veratridine on Na_V_1.7 and NaChBac to probe for conserved interaction mechanisms. The effect of aconitine (7 µM) on Na_V_1.7 current was highly voltage dependent. This can be appreciated in the current traces shown in Fig. 3B (top left), where the current elicited by a depolarizing pulse from − 100 to − 50 mV was increased, whereas the current induced by a pulse from − 100 to − 20 mV was decreased by aconitine (Fig. 3B, top left). The I–V plots of Fig. 3B (middle panel) illustrates the effects of aconitine on current magnitude across a wide range of test potentials, with enhancement observed for voltages below − 25 mV, robust inhibition at more depolarized potentials and a negative shift in the peak of the I-V relationship. Aconitine also shifted the G-V and SSI relationships for Na_V_1.7 channel currents (Fig. 3B, top). Intriguingly, inhibition of peak NaChBac channel currents was voltage-independent and was not accompanied by a shift in the I–V, G–V or SSI relationships (Fig. 3B, bottom). Aconitine is also a more potent inhibitor of NaChBac (IC_50_ = 1.3 µM) than of Na_V_1.7 (IC_50_ = 7.4 µM) channels (Supplemental Fig. 2. In summary, although aconitine inhibits both Na_V_1.7 and NaChBac, it only alters the gating of the Na_V_1.7 channels.

Veratridine inhibited both Na_V_1.7 and NaChBac channels at the test concentration of 7 µM (Fig. 3C, left and middle panels), but differentially affects their gating. In contrast to aconitine, veratridine primarily interfered with the inactivation gating of Na_V_1.7, causing a leftward shift of the SSI relationship with almost no effect on the G–V relationship (Fig. 3C, top right). Although veratridine enhanced the voltage dependence of inactivation, it prevented channels from fully inactivating, manifested as an increase in the sustained inward Na^+^ current measured at the end of a test pulse (Fig. 3C, top left). Conversely, while veratridine accelerated NaChBac inactivation (Fig. 3C, bottom left), it did not alter the voltage dependence of SSI (Fig. 3C, bottom right). Furthermore, veratridine induced a depolarizing shift in the G–V relationship of NaChBac. In summary, although veratridine blocks peak Na^+^ current of both channel types, it differentially modulates their gating, specifically altering SSI of Na_V_1.7 and inhibiting activation of NaChBac.

### F224 in the NaChBac channel is critical for its interaction with aconitine and veratridine

**Figure 4:**
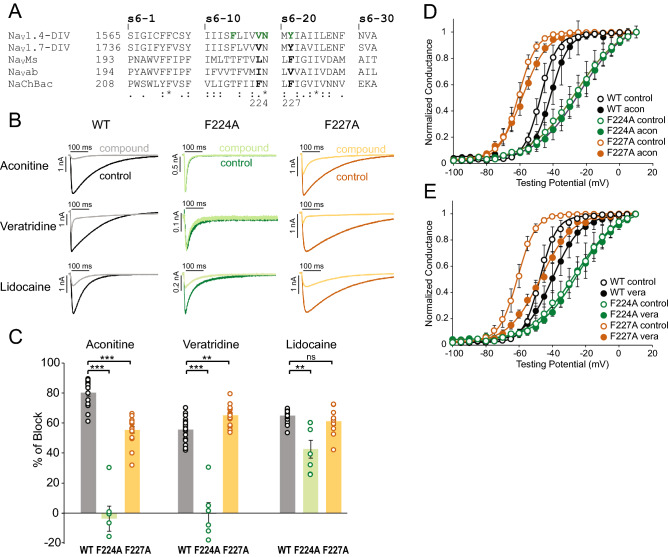
Identification of key interacting residues for aconitine and veratridine in NaChBac. (**A**) Sequence alignment of the S6 transmembrane segments of S6 of Na_V_1.4, Na_V_1.7 DIV, Na_V_Ms, Na_V_Ab, and NaChBac. The residues that were previously shown to be important for site-2 toxin batrachotoxin (BTX) in Na_V_1.4^35^ are labeled in green. The location of F224 and F227 residues of NaChBac are noted. (**B**) Representative current traces for WT NaChBac (left), F224A NaChBac (middle) and F227A NaChBac (right). Current traces recorded before and after the application of aconitine, veratridine, and lidocaine are shown. (**C**) Summary of percentage of peak current block induced by aconitine, veratridine, and lidocaine for WT, F224A and F227A NaChBac channels. Each data point represents recordings from one cell. Bars indicate mean ± S.E.M. for all cells. (**D**) G–V relationships for WT, F224A and F227A NaChBac channels before and after application of aconitine. (**E**) G–V relationships for WT, F224A and F227A NaChBac channels before and after application of veratridine.

To gain a better understanding of the interaction mechanisms between NaChBac and aconitine or veratridine, we used site-directed mutagenesis to probe for the potential location of their binding sites. Based on the sequence alignment of inner pore residues of Na_V_1.4, Na_V_1.7, Na_V_Ms, Na_V_Ab and NaChBac, we focused on several key residues that were previously shown to be important for site-2 toxin binding in human Na_V_1.4 channel^34,35^ (Fig. 3A). We mutated the conserved residues in NaChBac. We tested four NaChBac mutant channels, each containing one of the following point mutations: F221A, F224A, N225K, or F227A. Among them, F221A and N225K mutant channels did not functionally express (Supplement Fig. 3). F224A exhibited a lower peak current, a much faster rate of inactivation (Fig. 4B, Supplement Fig. 3) and an altered G–V relationship (rightward shifted with reduced slope; Fig. 4D) compared to WT NaChBac. F227A exhibited a hyperpolarizing shift in the G–V curve compared to WT channels (Fig. 4D). Aconitine (7 µM) caused about 80% inhibition of WT channel currents but had no effect on F224A channel currents and a reduced effect on F227A channels (Fig. 4B, C). The voltage dependence of activation (G–V curves) was not significantly altered by aconitine for any of the three channels (Fig. 4D). Veratridine (7 µM) inhibited peak currents of WT channels by 55.6 ± 2.0%, F227A channels by 65.1 ± 1.7%, but caused minimal or no block of F224A channels (Fig. 4B, C). Veratridine did not alter the G–V relationship of F224A channels, but caused a larger depolarizing shift in the G–V curve of F227A channels (ΔV_1/2_ = 20.1 ± 2.3 mV, WT ΔV_1/2_ = 7.7 ± 1.8 mV, p = 0.004) (Fig. 4E), further suggesting an enhanced effect of veratridine in the presence of the F227A mutation. Although both aconitine and veratridine interact strongly with F224, they may possess different spatial orientations within the pore, resulting in distinct interactions with the neighboring residue F227. We also tested how the two NaChBac mutations affected LA binding by assessing lidocaine blockade (Fig. 4B, C). We observed a decreased but still robust block by lidocaine of F224A channels, and no change in block of F227A channels.

## Methods and materials

### Cell lines

pcDNA3.1 (−) Hygro vector containing NaChBac or mutant cDNA was transfected into HEK 293 cells with Lipofectamine 2000 (Thermo Fisher, Waltham, MA). The transfected cells were cultured with selection antibiotics for a week, then single colonies were isolated and used for expansion. 24 colonies were tested with SyncroPatch 768PE for NaChBac expression. The colony with the highest expression was further expanded and used for characterization in this study. CHO cells stably expressing human Na_V_1.7 channels were constructed as described previously^36^. Cell lines were cultured in high glucose DMEM (HEK 293) or Ham’s F12 for (CHO), supplemented with 10% FBS, 2 mM L-glutamine and antibiotics in 5% CO_2_ at 37 °C.

### Electrophysiology recordings and compound applications

SyncroPatch 768PE (Nanion, Munich, Germany) was used to perform all automated patch clamp experiments. Chips with medium resistance (5-8MΩ) were used. The intracellular recording solution contained (in mM): 50 CsCl, 60 CsF, 10 NaCl, 20 EGTA and 10 HEPES (pH 7.2, osmolarity 285 mOsm), and extracellular recording solution contained (in mM): 80 NaCl, 60 NMDG, 4 KCl, 2 CaCl_2_, 1 MgCl_2_, 5 Glucose and 10 HEPES (pH 7.4, osmolarity 300 mOsm). Whole cell recordings were performed as previously described^12,36^. Series resistance compensation was set to 80%. Cell catching, sealing, whole-cell configuration formation, control solution application, recording, compound application and recording were performed sequentially. The holding membrane potential (V_m_) for all experiments was set at − 120 mV unless otherwise noted. For current–voltage (IV) relationship recordings, Na_V_1.7 and NaChBac channels were elicited by depolarizing voltage steps from − 80 mV to + 60 mV (5 mV increments) for 50 ms and 500 ms, respectively. Steady-state inactivation (SSI) protocol were performed by preconditioning V_m_ from − 120 to + 20 mV (5 mV increments) for 500 ms (Na_V_1.7) or 5 s (NaChBac), then followed with stimulation pulse at − 10 mV for 20 ms (Na_V_1.7) and 200 ms (NaChBac). For compound tonic block testing, a pulse from − 120 to − 20 mV was applied every 2 s, 5 min before and 20 min after applying the compound. All compounds were purchased from Alomone Labs (Israel), except compounds G0766 and G4936 were produced in house at Genentech. Small molecule compounds and water-soluble toxins were reconstituted in DMSO and extracellular recording solution, respectively. For compounds that were reconstituted in DMSO, control solution containing the same amount of DMSO was used. For small peptide toxins, 0.1% BSA was added to recording solutions to prevent non-specific binding during perfusion.

### Data analysis and statistics

Data were analyzed using Clampfit (v10; Molecular Devices), MATLAB (R2018b; MATLAB), and Excel (Microsoft). G–V and SSI curves were fitted to a Boltzmann function: y = 1/(1 + exp [(V − V_1/2_)/k]). Statistics for comparison of data recorded between before and after compound administration were performed using a paired Student t-test (Microsoft Excel). One-way ANOVA was used to compare drug block for different NaChBac mutant channels. Data are presented as mean ± SEM, from 5–20 cells.

## Discussion

Bacterial and mammalian sodium channels are separated by hundreds of millions of years of evolution. High resolution structures of bacterial Na_V_ channels have provided significant insights into the molecular basis of Na^+^ channel gating and ion selectivity. However, the molecular basis for the interactions between bacterial Na_V_ channels with pharmacological agents has remained understudied. Here, we systematically tested 39 eukaryotic Na_V_ channel-modulators and discovered intriguing similarities and differences in their interactions with Na_V_1.7 and NaChBac channels. Our study indicates that the divergence in pharmacology is determined by structural discrepancies, as well as by differences in gating dynamics between NaChBac and Na_V_1.7 channels.

Among a panel of 16 LA compounds known to bind to the pore of mammalian Na_V_ channels, 11 compounds exhibited robust block of NaChBac, whereas 5 compounds exhibit minimal effects on NaChBac when applied extracellularly, including three highly hydrophobic compounds (carbamazepine, oxcarbazepine and Co102862). It was proposed that these compounds access their binding site by diffusion from the membrane lipid bilayer into the inner pore^37^. This proposed hydrophobic diffusion pathway has since been identified as lateral fenestrations in the Na_V_Ab channel structure^6,20^. In supporting the relevance of this pathway to drug block, single residue mutations that decrease the size of fenestrations showed graded effects on resting-state block by the LAs flecainide, lidocaine, and benzocaine^20^. Compared to their robust effect on Na_V_1.7, the relatively bulky tricyclic compounds, such as Co102862 had minimal effect on NaChBac. It is possible that Co102862 preferentially binds to the inactivated state of Na_V_ channel (not present in NaChBac)^38^. Alternatively, the NaChBac fenestrations are too small to allow access of this compound to its inner pore.

Given that VSD-targeting toxins exhibit high specificities on mammalian Na_V_ channels, it is not unexpected that only 4 out of 15 toxins were able to modulate the NaChBac channel. From further assessment of GsAF-I and BDS-I, we observed that both toxins shifted the voltage dependence of inactivation of Na_V_1.7, but in opposite directions. In mammalian Na_V_ channels, the conformation of VSD of Domain IV (DIV-VSD) tightly regulates channel inactivation^27,39,40^. Inhibition of DIV-VSD activation (rightward shift) usually hinders inactivation, while leftward shift in voltage-dependence of DIV-VSD activation often promotes inactivation^14,41^. Therefore, it is likely that both GsAF-I and BDS-I interact with the DIV-VSD in the Na_V_1.7 channel, but in a different manner. In contrast to four distinct VSDs of Na_V_1.7 that contribute uniquely to channel gating, NaChBac has four symmetrical VSDs that equally contributes to gating. In NaChBac, although both GsAF-I and BDS-I inhibit peak Na^+^ currents, they have drastically different effects on channel gating: BDS-I caused inhibited channel activation (rightward shift of G-V curve), while GsAF-I had no significant effect. This data suggest that GsAF-I and BDS-I may have different affinities for VSDs at distinct conformational states, a mechanism that was demonstrated in ProTx-II modulation of Na_V_1.7^12^. Intriguingly, we observed that compounds that promote or stabilize the inactivated state in Na_V_1.7, including LAs, GsAF-I and aconitine, induced a small leftward shift or no effect on the G-V relationship of NaChBac. Conversely, compounds that inhibit inactivation in Na_V_1.7, such as BDS-I and veratridine, also induced significant rightward shifts in the G–V curves of NaChBac. These findings suggest a conservation between the DIV-VSD of the mammalian Na_V_ channels and the VSD of NaChBac in the toxin binding mechanism. Further, as NaChBac has symmetrical and simple VSD to pore coupling, by observing VSD-toxins’ effect on NaChBac G–V, we can speculate on their interactions with the mammalian Na_V_ channels’ DIV-VSDs.

Aconitine and veratridine are site-2 neurotoxins, defined by their activator activity resulting from binding to the intracellular pore of mammalian Na_V_ channels^33,34^. We demonstrated that both aconitine and veratridine bind to the F224 residue on NaChBac. Aconitine blocks peak current without affecting voltage-dependence of channel activation, while veratridine blocks peak current, speeds up inactivation and shifts activation to the depolarized direction. Interestingly, it was reported recently that BTX also binds to the F224 residue on NaChBac^42^. However, it causes hyperpolarization of activation and prevents deactivation of the channel^42^. Therefore, although the putative binding sites for aconitine, veratridine and BTX may overlap to some extent, their interaction with the inner pore results in distinct gating effects on NaChBac. Small molecules, such as lidocaine and PI1, bind to the Threonine residue (T220 in NaChBac) adjacent to the lipid-facing fenestration, as resolved in the Na_V_ab and Na_V_Ms structures^20,43^. Comparing to the small molecule binding sites, our findings suggest that aconitine and veratridine seem to occupy a lower position in the cavity, underlying their distinct modulation of NaChBac gating properties compared to small molecules. Notably, despite the four-fold symmetry of the NaChBac channel, due to the steric hindrance, pore-modulating compounds are likely to only bind to one subunit^20,43^, resulting in an asymmetric drug conformation in the pore, which further explains why site-2 toxins exert differential gating effects although sharing a common binding site.

Our current study also provides insights into organism-specific drug discovery. Bacterial ion channels are fundamental to the survival and function of bacteria, in terms of maintaining ion and pH homeostasis, controlling cell mobility, and cellular communications^44–46^. As a result, bacterial ion channels have emerged as new target for developing new antibiotics to combat the problem of multi-drug resistance (MDR)^47^. Here we identified a broad selection of compounds that modulate NaChBac function. Particularly, compounds such as aconitine exhibit higher potency on NaChBac compared to Na_V_1.7. These compounds and their related mechanisms can potentially be further explored for developing new antibiotics targeting the bacterial Na_V_ channels.

## Supplementary information

Supplementary information
