# Supplementary material for: Conservation and divergence in NaChBac and NaV1.7 pharmacology reveals novel drug interaction mechanisms
Source: Sci Rep. 2020 Jul 1;10:10730. doi: 10.1038/s41598-020-67761-5 (PMC7329812; doi:10.1038/s41598-020-67761-5)
Supplement: Supplementary file 1 — Supplementary information [file 41598_2020_67761_MOESM1_ESM.docx]

**Supplement Figure 1: Percentage of block in NaChBac by compounds in four classes. Red bar indicates a “hit” and blue bar indicates a “miss”.** A. LA site-binding small molecules. B. VSD-targeting toxins. C. Pore-targeting toxins. D. Isoform-specific molecules.


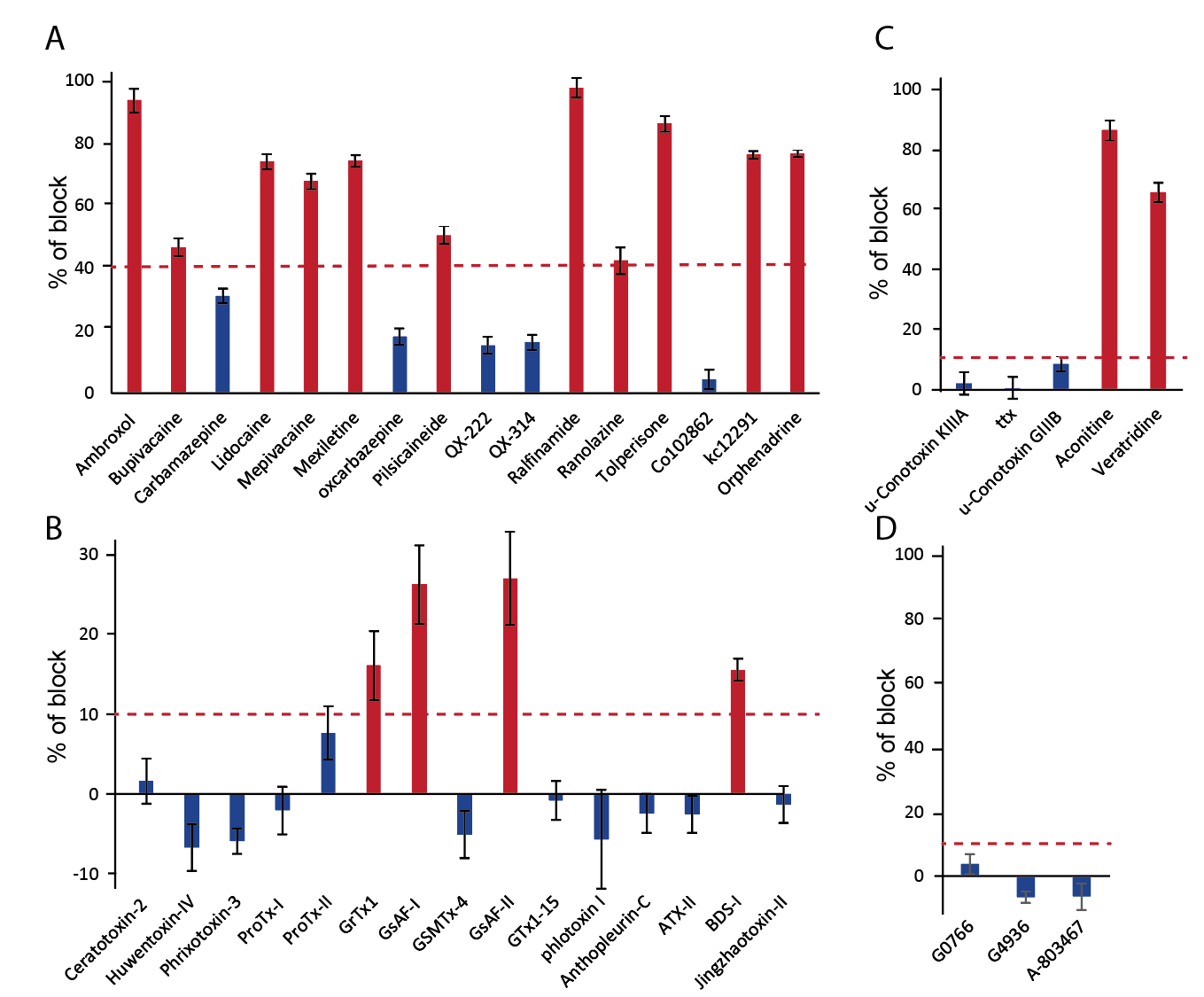


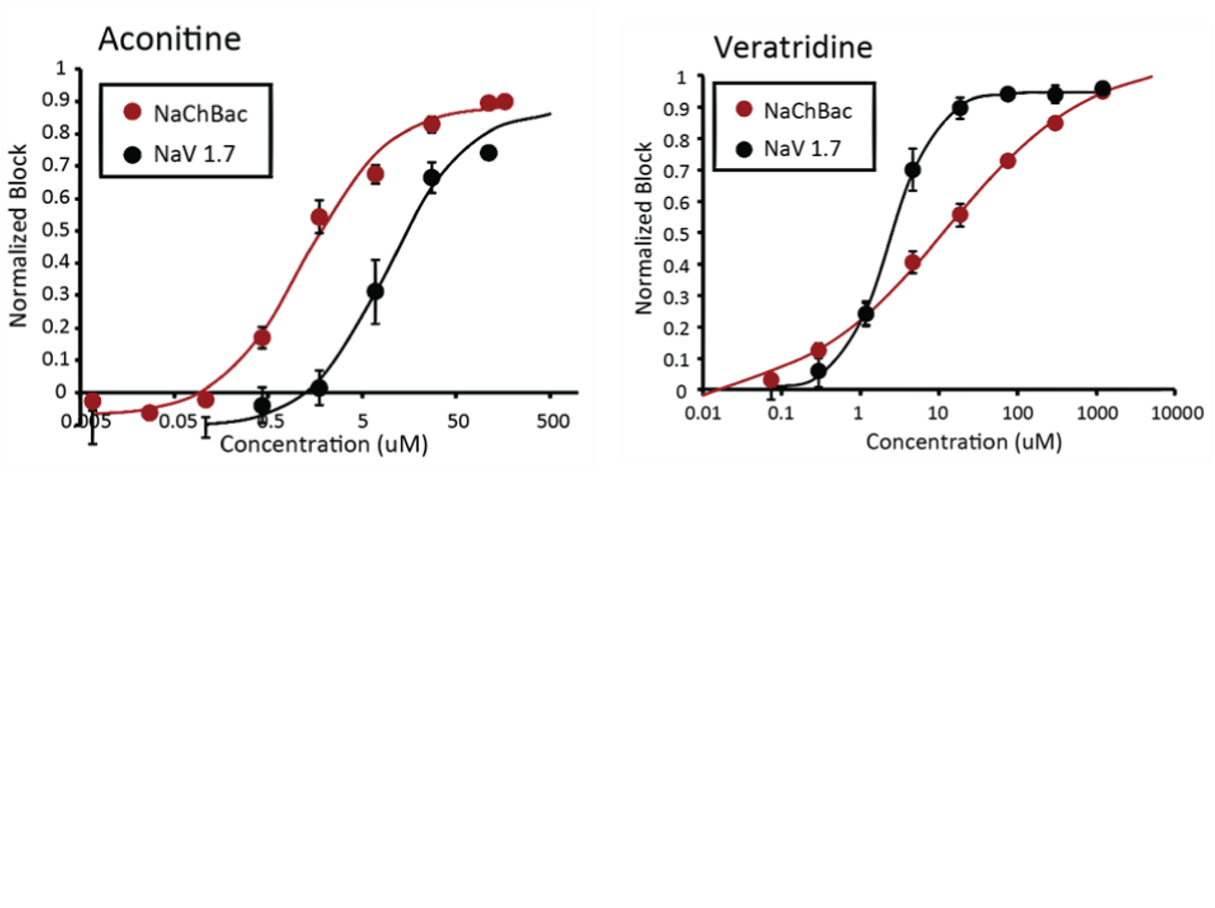
**Supplement Figure 2: Dose-response curves of aconitine and veratridine in NaChBac (red) and Na_V_1.7 (black).** Aconitine IC_50_ for NaChBac is 1.3uM, Hill slope is 1.0. Aconitine IC_50_ for Na_V_1.7 is 7.4uM, Hill slope is 1.6. Veratridine IC_50_ for NaChBac is 8.1uM, Hill slope is 0.3. Veratridine IC_50_ for Na_V_1.7 is 2.0uM, Hill slope is 1.5.

**Supplement Figure 3: Representative currents (A) and current amplitudes (B) for F221A, F224A, N225K, and F227A mutants of NaChBac channel.**

**
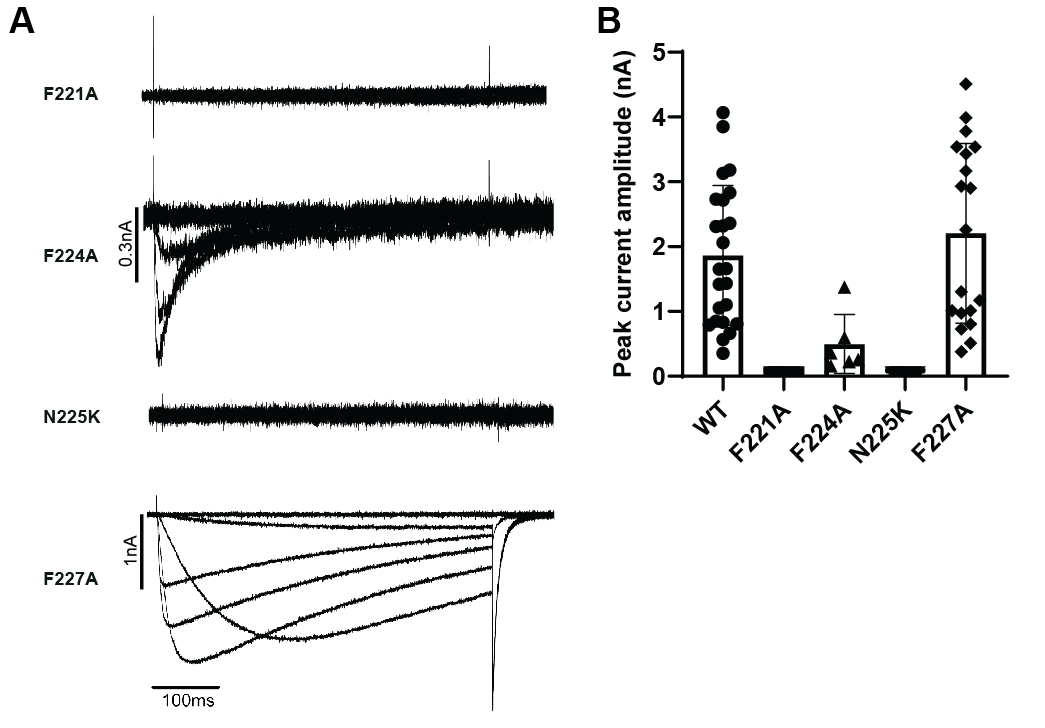
**

**Supplement table I: Screening results of all compounds blockade of the NaChBac channels.** The percentage of block was reported from groups of 6-15 cells. The testing concentrations are shown as in table.

| Compounds | Testing concentrations (uM) | % of block in NaChBac |
| --- | --- | --- |
| LA site-binding small-molecules | | |
| Ambroxol | 150 | 93.6±3.8 |
| Bupivacaine | 23 | 46.6±2.9 |
| Carbamazepine | 1200 | 31.1±2.4 |
| Lidocaine | 300 | 74.1±2.3 |
| Mepivacaine | 225 | 67.7±2.5 |
| Mexiletine | 150 | 74.2±1.8 |
| oxcarbazepine | 300 | 18.2±2.5 |
| Pilsicaineide | 300 | 50.5±2.7 |
| QX-222 | 1500 | 15.3±2.7 |
| QX-314 | 1500 | 16.2±2.6 |
| Ralfinamide | 120 | 97.7±3.0 |
| Ranolazine | 75 | 42.5±4.3 |
| Tolperisone | 300 | 86.2±2.5 |
| Co102862 | 50 | 4.4±3.1 |
| kc12291 | 50 | 76.3±1.1 |
| Orphenadrine | 100 | 76.6±1.1 |
| VSD-targeting toxins | | |
| Ceratotoxin-2 | 0.4 | 1.6±2.9 |
| Huwentoxin-IV | 0.3 | -6.7±2.9 |
| Phrixotoxin-3 | 0.25 | -5.9±1.6 |
| ProTx-I | 0.1 | -2.0±3.0 |
| ProTx-II | 0.08 | 7.7±3.3 |
| GrTx1 | 1 | 16.2±4.3 |
| GsAF-I | 1 | 26.2±4.9 |
| GSMTx-4 | 2 | -5.1±3.0 |
| GsAF-II | 3 | 27.1±5.8 |
| GTx1-15 | 1 | -0.8±2.4 |
| phlotoxin I | 1 | -5.7±6.2 |
| Anthopleurin-C | 0.05 | -2.4±2.4 |
| ATX-II | 0.05 | -2.5±2.3 |
| BDS-I | 0.2 | 15.6±1.3 |
| Jingzhaotoxin-II | 0.25 | -1.3±2.3 |
|  | | |
| Pore-targeting toxins | | |
| u-Conotoxin KIIIA | 0.5 | 2.1±3.7 |
| ttx | 1 | 0.5±3.6 |
| u-Conotoxin GIIIB | 0.5 | 8.6±2.4 |
| Aconitine | 6 | 86.3±3.4 |
| Veratridine | 50 | 65.6±3.2 |
| Isoform-specific molecules | | |
| G0766 | 1 | 3.7±3.1 |
| G4936 | 1 | -6.5±1.8 |
| A-803467 | 1 | -6.3±4.1 |
